# 3D printed-electrospun PCL/hydroxyapatite/MWCNTs scaffolds for the repair of subchondral bone

**DOI:** 10.1093/rb/rbac104

**Published:** 2022-12-14

**Authors:** Yanyan Cao, Lei Sun, Zixian Liu, Zhizhong Shen, Wendan Jia, Peiyi Hou, Shengbo Sang

**Affiliations:** College of Information Science and Engineering, Hebei North University, Zhangjiakou 075000, China; Shanxi Key Laboratory of Micro Nano Sensors & Artificial Intelligence Perception, College of Information and Computer, Taiyuan University of Technology, Taiyuan 030024, China; Shanxi Key Laboratory of Micro Nano Sensors & Artificial Intelligence Perception, College of Information and Computer, Taiyuan University of Technology, Taiyuan 030024, China; Key Lab of Advanced Transducers and Intelligent Control System of the Ministry of Education, Taiyuan University of Technology, Taiyuan 030024, China; Shanxi Key Laboratory of Micro Nano Sensors & Artificial Intelligence Perception, College of Information and Computer, Taiyuan University of Technology, Taiyuan 030024, China; Key Lab of Advanced Transducers and Intelligent Control System of the Ministry of Education, Taiyuan University of Technology, Taiyuan 030024, China; Shanxi Key Laboratory of Micro Nano Sensors & Artificial Intelligence Perception, College of Information and Computer, Taiyuan University of Technology, Taiyuan 030024, China; Shanxi Research Institute of 6D Artificial Intelligence Biomedical Science, Taiyuan 030031, China; Shanxi Key Laboratory of Micro Nano Sensors & Artificial Intelligence Perception, College of Information and Computer, Taiyuan University of Technology, Taiyuan 030024, China; Shanxi Research Institute of 6D Artificial Intelligence Biomedical Science, Taiyuan 030031, China; Shanxi Key Laboratory of Micro Nano Sensors & Artificial Intelligence Perception, College of Information and Computer, Taiyuan University of Technology, Taiyuan 030024, China; Shanxi Research Institute of 6D Artificial Intelligence Biomedical Science, Taiyuan 030031, China; Shanxi Key Laboratory of Micro Nano Sensors & Artificial Intelligence Perception, College of Information and Computer, Taiyuan University of Technology, Taiyuan 030024, China; Key Lab of Advanced Transducers and Intelligent Control System of the Ministry of Education, Taiyuan University of Technology, Taiyuan 030024, China

**Keywords:** additive manufacturing, 3D printing, electrospinning, subchondral bone

## Abstract

Osteochondral defect caused by trauma or osteoarthritis exhibits a major challenge in clinical treatment with limited symptomatic effects at present. The regeneration and remodeling of subchondral bone play a positive effect on cartilage regeneration and further promotes the repair of osteochondral defects. Making use of the strengths of each preparation method, the combination of 3D printing and electrospinning is a promising method for designing and constructing multi-scale scaffolds that mimic the complexity and hierarchical structure of subchondral bone at the microscale and nanoscale, respectively. In this study, the 3D printed-electrospun poly(ɛ-caprolactone)/nano-hydroxyapatites/multi-walled carbon nanotubes (PCL/nHA/MWCNTs) scaffolds were successfully constructed by the combination of electrospinning and layer-by-layer 3D printing. The resulting dual-scale scaffold consisted of a dense layer of disordered nanospun fibers and a porous microscale 3D scaffold layer to support and promote the ingrowth of subchondral bone. Herein, the biomimetic PCL/nHA/MWCNTs scaffolds enhanced cell seeding efficiency and allowed for higher cell–cell interactions that supported the adhesion, proliferation, activity, morphology and subsequently improved the osteogenic differentiation of bone marrow mesenchymal stem cells *in vitro*. Together, this study elucidates that the construction of 3D printed-electrospun PCL/nHA/MWCNTs scaffolds provides an alternative strategy for the regeneration of subchondral bone and lays a foundation for subsequent *in vivo* studies.

## Graphical abstract

**Figure:**
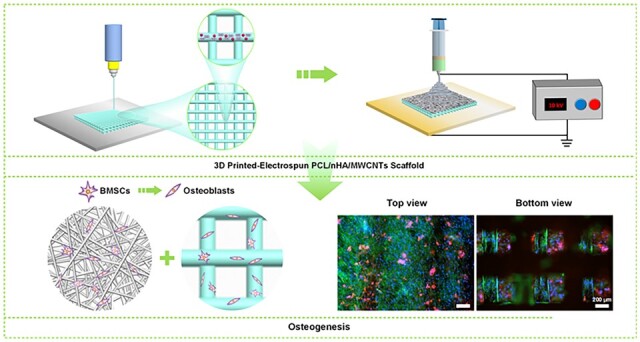

## Introduction

Osteochondral defect is a common complaint resulted from trauma or osteoarthritis, and its treatment is a major challenge in clinic [1, 2]. It is well known that the osteochondral complex consists of articular cartilage and subchondral bone, which are closely connected and well integrated. Subchondral bone is composed of a subchondral plate and trabeculae, which are critical for joint function, cushioning mechanical loads and providing nutrients to the cartilage [3, 4].Without the support of healthy subchondral bone, cartilage defects are difficult to repair [5]. However, over the past few decades, various biomimetic scaffolds have been developed for cartilage repair and considerable success has been achieved [6–8]. By comparison, the biomimetic scaffolds for subchondral bone repair have not become the keystone of research. Bone substitutes are also rarely assessed for subchondral bone defects. In clinical sense, the optimal preparation method, biomaterial and implant method of biomimetic subchondral bone scaffold will be helpful to the treatment of osteochondral defect [9].

For subchondral bone regeneration, the biomimetic scaffolds should not only possess excellent bioactivity and appropriate mechanical stability to match subchondral bone and mimic the extracellular matrix (ECM) of natural bone tissue, but also provide dual-scale anisotropy to guide the in-growth of bone tissue [10, 11]. Enormous advances in additive manufacturing technology can meet this need, allowing the fabrication of scaffolds with customized features and desirable properties that can be combined with multi-material structures [12]. Currently, bone scaffolds prepared by 3D printing have fully interconnected pore networks that facilitate the delivery of proteins, oxygen and nutrients, as well as the in-growth of bone tissue, which cannot be achieved with traditional techniques [13, 14]. Whereas, the pore size of 3D printed scaffolds is commonly limited to microscale, which adversely affects cell seeding efficiency [15]. In order to mimic ECM nanoscale fibers, alternative technologies are developed, such as electrospinning, which can provide nanoscale ECM-mimicking structures with high specific surface area and high porosity [16]. Electrospun nanofibers offer many adhesion sites for cell attachment and growth, thereby affecting cell morphology and activity, but their low mechanical properties limit their application [17, 18]. Thus, a new idea that combine 3D printing and electrospinning methods are proposed in this paper to give full play to their strengths and mitigate their weaknesses, which provide an effective and powerful preparation method to mimic the complexity and hierarchical structure of subchondral bone at the microscale and nanoscale, respectively.

The selection of biomaterials with desirable characteristics is also the key to the successful regeneration of subchondral bone. Due to its excellent mechanical properties and reasonable biodegradability, poly(ɛ-caprolactone) (PCL) has been broadly studied in bone tissue engineering [19]. Even so, the biological property of PCL can be enhanced by incorporating with other biomaterials [19, 20]. Hydroxyapatite (Ca_10_(PO_4_)_6_(OH)_2_) is a kind of bioactive ceramic material, which is the major inorganic component of natural bone matrix [21]. Because of its excellent biocompatibility and high bone conductivity, it has been extensively used in clinical bone transplantation [22]. Additionally, nano-hydroxyapatite (nHA) has also been reported to promote cell adhesion, proliferation and calcium deposition [23]. Meanwhile, new strategies for bone regeneration tend to combine sturdy biomaterials with smart biomaterials that respond to external stimuli, such as electrical stimulation, to speed up the healing process [24]. Carbon nanotubes (CNTs) are cylindrical nanostructures, available in the form of single-walled (SWCNTs) or multi-walled (MWCNTs), with remarkable mechanical and electrical properties [25–27]. CNTs possess unique size characteristics similar to those of fibrinogen (such as collagen) in bone, with a length–diameter ratio of more than 10^6^, which is similar to the size of triple-helix collagen fibers, making them particularly important for bone regeneration applications [28].Carboxyl or hydroxyl functionalized CNTs can enhance the biocompatibility, hydrophilicity, uniform dispersion and bonding with polymeric matrix [29, 30]. Interestingly, studies have shown that MWCNTs has a stronger ability to induce osteogenic differentiation of stem cells than Haps [31]. The possible mechanism was that MWCNTs aggregate more proteins, including specific osteoinductive proteins, thereby activating Notch signaling pathway. Therefore, we plan to prepare PCL/nHA/MWCNTs composite scaffolds to support the adhesion, proliferation and osteogenic differentiation of bone marrow mesenchymal stem cells (BMSCs).

This study developed an attractive method to fabricate 3D PCL/nHA/MWCNTs scaffolds using electrospinning combined with layer-by-layer 3D printing. It was hypothesized that such the structure and composition can satisfy both the physical (e.g. morphology, porosity and mechanical properties) and biological (e.g. the adhesion, proliferation, activity, morphology and osteogenic differentiation of BMSCs) requirements of the scaffold for subchondral bone regeneration. As expected, the 3D printed-electrospun PCL/nHA/MWCNTs scaffolds possessed excellent biocompatibility and presented the most positive effect on the osteogenic differentiation of BMSCs *in vitro*.

## Materials and methods

### Materials and reagents

PCL (Mw = 80 000), nano-hydroxyapatites with the particle size of 60–80 nm and short carboxyl-functionalized MWCNTs (5–10 nm in inner diameter, 10–20 nm in outer diameter, 0.5–2 µm in length and purity per cent of 95%) were purchased from Macklin Biochemical Co., Ltd. (Shanghai, China). Tetrahydrofurane (THF) and dimethylformamide (DMF) were purchased from Sigma-Aldrich (USA). Rat BMSCs were obtained from Procell (Wuhan, China). All cell culture reagents including Dulbecco’s modified eagle medium-F12 (DMEM-F12), fetal bovine serum (FBS), streptomycin/penicillin and trypsin were purchased from Solarbio (Beijing, China).

### Scaffold design and fabrication

**Figure 1.**
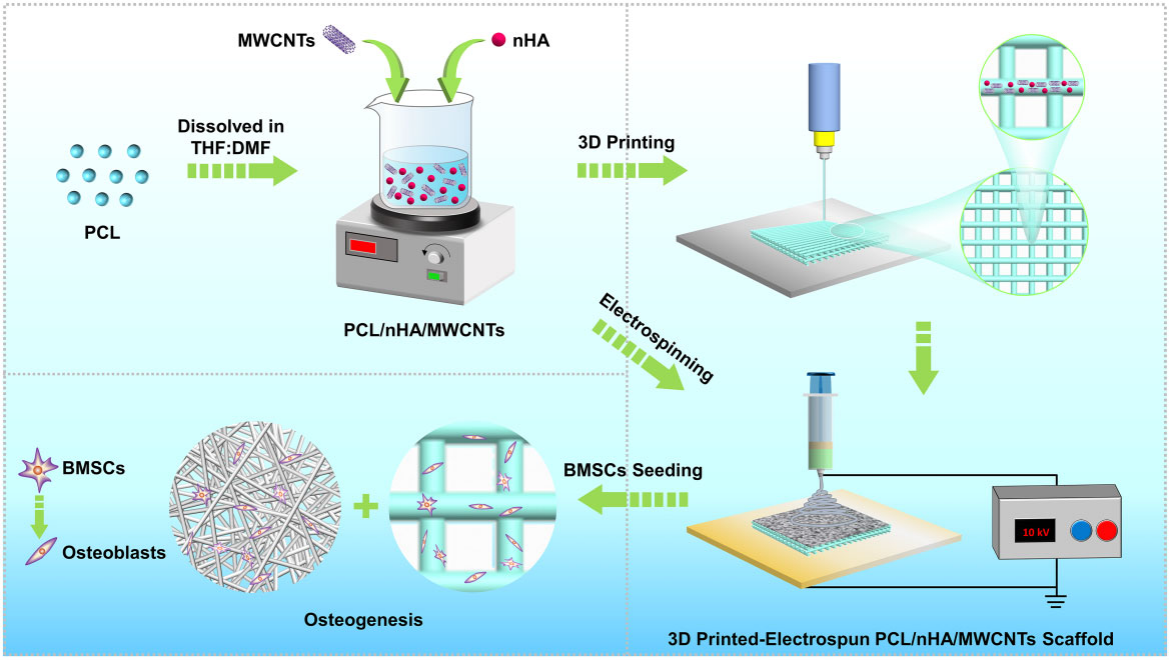
Schematic illustration of preparation of 3D printed-electrospun PCL/nHA/MWCNTs scaffold for bone regeneration.

The schematic of preparation of three-dimensional (3D) printed-electrospun PCL/nHA/MWCNTs composite scaffold was shown in Fig. 1. PCL pellets were dissolved in a 1:1 v/v mixture of THF:DMF to prepared PCL solutions, which was stirred magnetically at 40°C for 24 h. Next, a prespecified number of nHA and MWCNTs were dispersed into PCL solution by magnetic stirring for 24 h and followed sonicated in the cooling water circulation for 4 h, to prepare homogeneous PCL, PCL/nHA (90/10 wt%) and PCL/nHA/MWCNTs (89/10/1 wt%) composite solutions.

The dual-scale PCL/nHA/MWCNTs composite scaffold was constructed using an extrusion-based 3D printing system (RKJ3DP-4A-02, Recongene Biomedical Technologies, Ltd., Nanjing, China) combined with an electrospinning system (HY-01, Yonker Huayuan, Langfang, China). In this study, scaffolds were designed by computer-aided design (CAD) software, with parameters of four layers, filament diameter of 200 μm, filament spacing of 500 μm and layer thickness of 200 μm. The 3D-printed scaffolds with 0°/90° lay-down pattern were printed by extruding composite inks though a nozzle with an inner diameter of 300 μm. The temperatures of the upper and lower printing chamber were 75°C and 125°C, respectively. The pneumatic pressure was 7 bar, the temperature of glass platform was 50°C, the printing speed was 50 mm/s and the control ratio of extrusion was 3%. Subsequently, the scaffolds were cooled to room temperature after printing, removed from the glass plate, and electrospun using an electrospinning system to create the PCL/nHA/MWCNTs electrospun membranes with thickness of approximately 0.1 mm. The composite solution was fed into 5 ml standard syringes equipped with a 22G blunted stainless steel needle. The optimized electrospun parameters were as follow, the voltage was 10 kV, flow rate was 1 ml/h, and the distance between spinneret and collector was 15 cm. The fibers were collected on 3D-printed PCL/nHA/MWCNTs scaffold pasted on the aluminium foil and stored at room temperature for 24 h in a vacuum drier to remove residual solvents. Finally, the 3D printed-electrospun scaffolds were cut into cylinders with diameter of 14 mm and sterilized with 60 Co-γ-ray radiation to facilitate cell culture. Additionally, pure 3D printed-electrospun PCL and PCL/nHA scaffold were fabricated as control.

### Scaffold morphology

The macroscopic morphology of 3D printed-electrospun scaffolds were observed by using a super depth-of-field (DOF) optical microscope (DSX1000, Olympus, Japan). The scanning electron microscopy (SEM) and energy dispersive spectroscopy (EDS) images of scaffolds were analyzed with Hitachi SU8010 SEM (Japan). Dry scaffolds were sputtered with gold for 60 s before scanning, and the accelerating voltage was 5 kV. Besides, the images were analyzed by Image J software. In particular, the diameter of electrospun fibers (*n* = 100), the filament width (*n* = 30) and pore size of 3D printed scaffolds were evaluated by random selection and calculation.

### Porosity

The porosity of the scaffold was determined by ethanol immersion method [32]. Briefly, the dry weight (*W*_S_) of each scaffold was first measured, and the scaffold was then immersed in ethanol for 30 min to guarantee thorough infiltration into the pores. The weight of the gravity bottle filled full with ethanol was labeled as *W*_E_, the weight of the gravity bottle filled with ethanol and the scaffold was labeled as *W*_ES_, and the weight after removing the saturated scaffold was recorded as *W*_E’_. Thus, the calculation equation of porosity is as follows.

(1) $$\mathrm{Porosity}\;\left(\%\right)=\left(W_{\text{ES}}-W_{E’}-W_{S}\right)/\left(W_{E}-W_{E’}\right)\times 100\%$$

### Mechanical analysis

Cylindrical scaffolds (14 mm in diameter and 1.2 mm in height) were fabricated for compressive performance evaluation using a mechanical test machine (Instron 3343, USA) furnished with a 1 kN load cell. All scaffolds were tested at a compression rate of 0.2 mm/min. The compressive modulus was obtained from the slope of the initial linear regions (5–20%) of the compressive stress-strain curve.

### Cytocompatibility analysis

#### BMSCs culture and seeding

Cylindrical scaffolds were irradiated with 60 Co-γ rays and rinsed with PBS at least twice. BMSCs were cultured in DMEM-F12 with additional 10% (v/v) FBS and 1% (v/v) streptomycin/penicillin, and seeded onto sterilized scaffolds kept in 24-well plates with a cell density of 4 × 10^4^ per sample. All cells-scaffolds were cultured at 37°C in a 95% humidity and 5% CO_2_ atmosphere. For osteogenic differentiation of BMSCs, no osteogenic differentiation medium was used in the experiment.

#### Cell viability and proliferation

Cell viability was evaluated by double-color fluorescence staining with a LIVE/DEAD Viability/Cytotoxicity assay (BestBio, Shanghai, China) at day 1, 3 and 7. Based on the manufacturer’s instruction, the scaffolds were submerged in LIVE/DEAD staining solution at 37°C for 2 h, and then photographed with a cell imaging reader (Cytation5, BioTek, USA) to distinguish living cells (green) and dead cells (red).

Cell proliferation on scaffolds (*n* = 3) was assessed by Cell Counting Kit assay (CCK-8, Abbkine, Wuhan, China) as previous reported methods [33]. At the predetermined time (1, 3, 5 and 7 days), the scaffolds were gently rinsed in PBS and immersed in fresh culture medium with additional 10% CCK-8 solution at 37°C for 2 h. The absorbance (450 nm) was determined by a microplate reader (Cytation5, BioTek, USA). Cell proliferation rate (%) was calculated as follows.

(2) $$\mathrm{Cell proliferation}\left(\%\right)=\left(OD_{D}-OD_{1}\right)/OD_{1}\times 100\%$$

where *OD*_1_ represents the absorbance on the first day and *OD*_D_ represents the absorbance at different points in time.

#### Cell morphology

Cell morphology and adhesion were determined using cytoskeleton staining and SEM. After 7 and 14 days of culture, BMSCs cultured on scaffolds were fixed with precooled 4% paraformaldehyde (PFA) for 20 min, then incubated in Fluorescent Dye 488-I Phalloidin (AmyJet Scientific, Wuhan, China) solution for 45 min to stain the f-actin cytoskeleton and in 4',6-diamidino-2-phenylindole (DAPI, Abcam) solution for 10 min to dye the nuclei. Subsequently, samples were observed with a cell imaging reader. For cell adhesion, BMSCs were cultured on the scaffolds for 14 days, then fixed with 2.5% glutaraldehyde for 3 h, dehydrated with graded ethanol (30, 50, 75, 80, 95 and 100% twice, 15 min for each), and dried with critical point drier. Finally, the images were obtained with SEM (SU8010, Hitachi, Japan) at 5 kV after gold sputter-coated.

### Osteogenic differentiation of BMSCs

#### Alkaline phosphatase (ALP) activity

To evaluate the early markers of osteogenic differentiation of BMSCs, ALP staining and ALP activity determination were performed on days 7 and 14. Briefly, after washing gently, cells–scaffolds were incubated in BCIP/NBT ALP Color Development Kit (Beyotime, Shanghai, China) solution for 30 min at room temperature in darkness. Subsequently, samples were photographed by an optical microscope (Eclipse Ci Pol, Nikon) to visualize the ALP activity.

Additionally, an ALP Assay Kit (Beyotime) was used for quantitative determination of ALP activity. In brief, cells–scaffolds were washed twice, lysed with a cell lysis buffer, and centrifuged at 4°C at 6000 rpm for 10 min. Then the supernatant was extracted, para-nitrophenyl phosphate (pNPP) substrate was added and incubated at 37°C for 30 min. Meanwhile, BCA Protein Assay Kit (Beyotime) was used to measure total protein in supernatant. The absorbance of the resultant yellow and purple compound were determined at 405 and 562 nm with a microplate reader, respectively. The measured ALP concentration for each sample was then normalized to total protein concentration.

#### Evaluation of ECM mineralization

ECM mineralization of BMSCs was assessed by Alizarin Red S (ARS) Staining Kit (Beyotime) for osteogenesis after 7 and 14 days of culture. In short, cells–scaffolds were rinsed twice with PBS, fixed in 4% PFA for 20 min, and submerged in ARS solution for 30 min at room temperature. The unabsorbed dye was removed with PBS. Images were photographed by an optical microscope (Nikon) to visualize calcium deposits. Besides, the quantitative analysis was performed by elution of the adsorbed stain in 500 μl 10% cetylpyridinium chloride in 10 mM sodium phosphate (pH 7.0). The absorbance was read at 570 nm using a microplate reader.

#### Gene expression analysis

BMSCs were seeded onto the 3D printed-electrospun PCL and PCL/nHA/MWCNTs scaffolds for 7 and 14 days. Total RNA of BMSCs was extracted by TRIzol reagent (Invitrogen), and reverse-transcribed to complementary DNA with PrimeScriptTM RT Master Mix (TaKaRa). qRT-PCR was performed by Bio-Rad CFX 96 Touch Real-Time PCR Detection System with TB GreenTM Premix Ex TaqTM II (TaKaRa). The primer pair sequences for osteogenic differentiation genes are summarized in Table 1, including ALP, collagen I (COL1), runt-related transcription factor 2 (RUNX2), osteocalcin (OCN) and osteopontin (OPN). The relative expression levels of osteogenic marker genes were normalized to the housekeeping gene (glyceraldehyde-3-phosphate dehydrogenase, GAPDH), and calculated by the 2^−ΔΔCt^ method using PCL group on day 7 as the reference group.

**Table 1.** Primer pair sequences for osteogenic differentiation genes

Gene	Primer sequences	
	Forward (5' to 3')	Reverse (5' to 3')
GAPDH	GCCACATCGCTCAGACACC	CCCAATACGACCAAATCCGT
ALP	AGCAGCATCTTACCAGTTGTGTCTC	AAGTAGTTCACATCCTGCGGTTCAG
COL1	TGTTGGTCCTGCTGGCAAGAATG	GTCACCTTGTTCGCCTGTCTCAC
RUNX2	CTTCGTCAGCGTCCTATCAGTTCC	TCCATCAGCGTCAACACCATCATTC
OCN	GGACCCTCTCTCTGCTCACTCTG	ACCTTACTGCCCTCCTGCTTGG
OPN	GACGATGATGACGACGACGATGAC	GTGTGCTGGCAGTGAAGGACTC

#### Osteogenic immunofluorescence staining

After 14 days of culture, the expression of the osteogenic marker proteins secreted by BMSCs on the 3D printed-electrospun PCL and PCL/nHA/MWCNTs scaffolds was observed by immunofluorescence staining. BMSCs-seeded scaffolds were fixed with 4% PFA for 1 h, treated with 0.1% Triton X-100 for 10 min and 1% BSA for 1 h. Then, the samples were incubated with primary antibody solution for ALP (1:100, Abcam), COL1 (1:50, ABclonal), OPN (1:100, Bioss) or OCN (1:100, Abcam) at 4°C overnight. Afterward, the samples were incubated with Cy3-conjugated goat anti-rabbit IgG secondary antibody for 2 h at room temperature. Finally, the cytoskeleton and DAPI staining were performed and the samples were observed with a cell imaging reader.

### Statistical analysis

All data were replicated at least three times, and showed as mean ± standard deviation. One-way ANOVA with a *post hoc* test was conducted for statistical analysis, *P <* *0.05, **P <* *0.01 and ***P <* *0.001 were considered statistically significant.

## Results and discussion

### Characterization of the biomimetic subchondral bone scaffold

**Figure 2.**
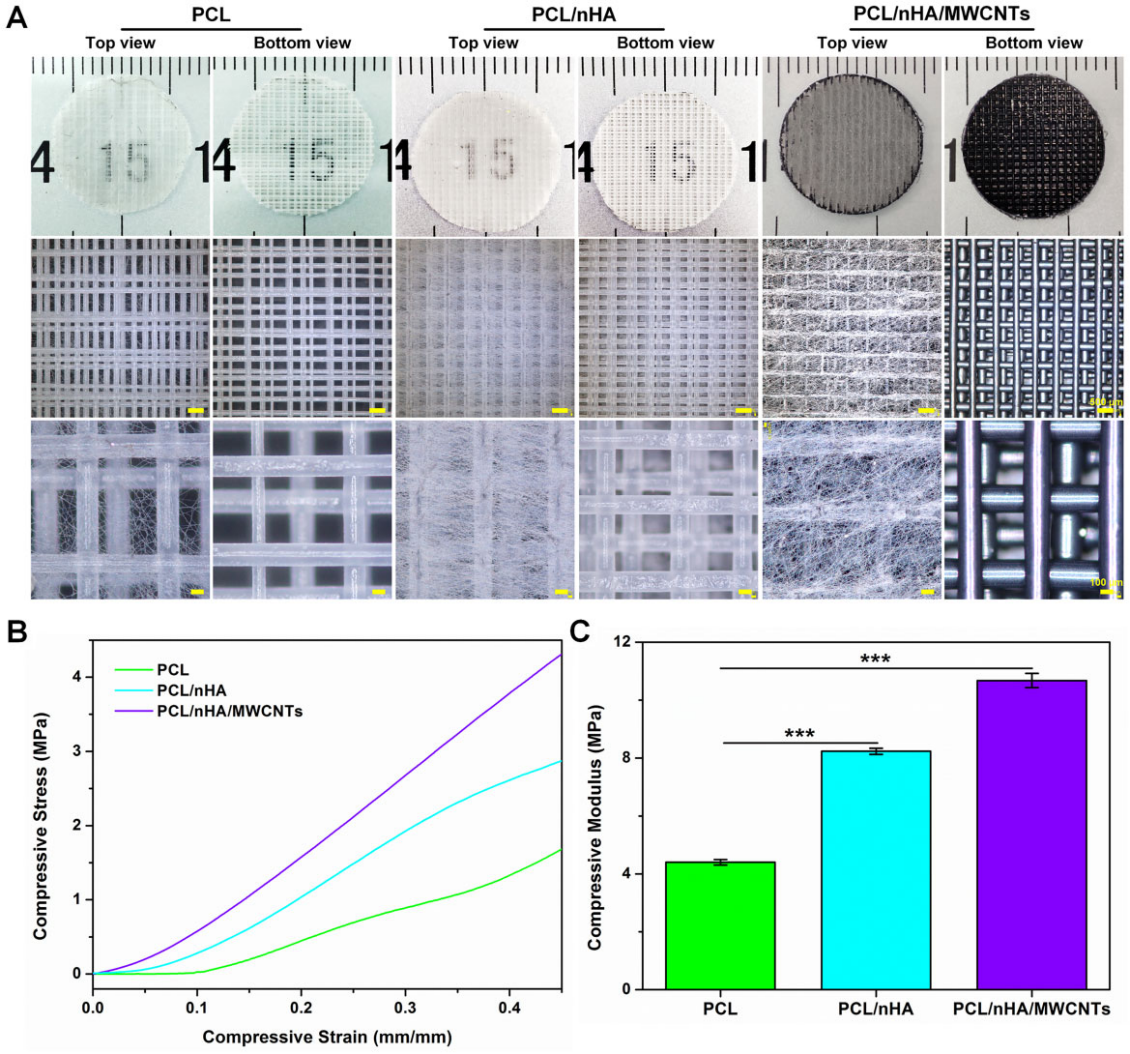
(**A**) Optical images, (**B**) compressive stress–strain curves and (**C**) compressive modulus of the 3D printed-electrospun PCL, PCL/nHA and PCL/nHA/MWCNTs scaffolds.

In this study, novel biomimetic subchondral bone scaffolds were successfully prepared. The microscale PCL/nHA/MWCNTs scaffold was created via 3D printing, and then micro-nano structure surface on the PCL/nHA/MWCNTs scaffold was constructed by electrospinning (Fig. 1). As the control group, pure PCL scaffolds and PCL/nHA scaffolds were prepared by the same procedure. The scaffolds were laid in 0°/90° pattern to ensure their 3D porous structure, and exhibited well-designed macroporous morphology (Fig. 2A). The 3D-printed PCL/nHA/MWCNTs scaffolds appeared black due to the presence of MWCNTs, while the PCL and PCL/nHA scaffolds appeared white. After electrospinning, micro-nano structures were uniformly dispersed on the surface of the 3D-printed scaffolds, and the fibers were smooth without beads (Fig. 2A). PCL/nHA/MWCNTs fibers were gray, and the density of PCL/nHA/MWCNTs and PCL/nHA fibers was higher than that of PCL fibers.

**Figure 3.**
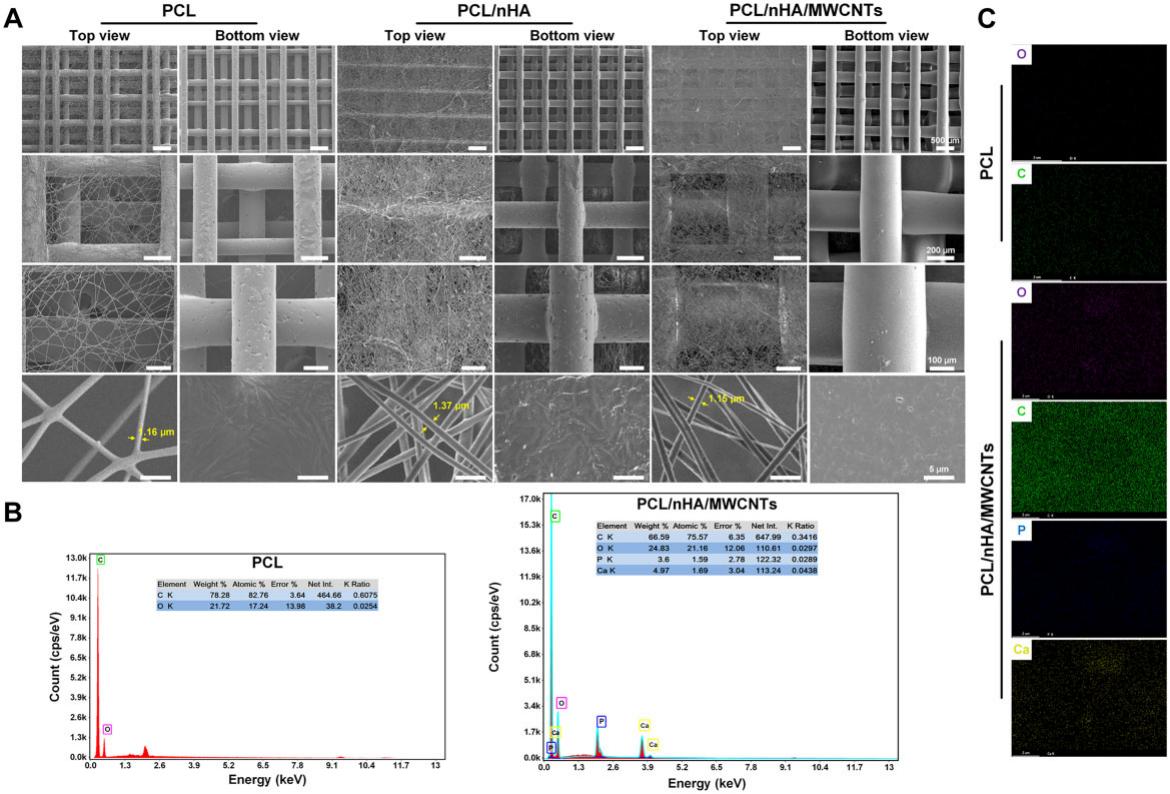
Characterization of the 3D printed-electrospun scaffolds. (**A**) Representative SEM images, (**B**) EDS spectrum and (**C**) elemental distribution mapping images of PCL and PCL/nHA/MWCNTs scaffolds, including oxygen (O), carbon (C), phosphorus (P) and calcium (Ca).

Moreover, the surface characteristics of the 3D printed-electrospun scaffolds were also revealed by SEM (Fig. 3A). The 3D-printed microscale scaffolds were well-defined and have a mesh structure. PCL filament width and the pore size were 200.07 ± 19.89 and 504.54 ± 117.50 μm, PCL/nHA filament width and the pore size were 209.20 ± 15.81 and 494.89 ± 41.03 μm, and PCL/nHA/MWCNTs filament width and the pore size were 239.45 ± 41.96 and 472.83 ± 59.79 μm, respectively (Table 2). The 3D-printed microscale scaffolds had a multi-layer structure that supports cell migration, allows cells to adhere to more areas, and facilitates the transfer of nutrients and metabolites. In the same electrospinning time, PCL/nHA/MWCNTs fibers had a slightly larger diameter and a slightly smaller mesh density than PCL/nHA and PCL, but the nanoscale electrospun fibers of all scaffolds showed porous and randomly guided fiber structures, mostly about 1 μm. The average diameter of PCL/nHA/MWCNTs fibers was 1.68 ± 0.62 μm, and the average diameter of PCL/nHA and PCL fibers was 1.74 ± 0.29 and 1.47 ± 0.38 μm, respectively (Table 2). This is because the intrinsic conductivity of MWCNTs leads to an increase in the stretching of PCL/nHA/MWCNTs nanofibers in the electromagnetic field during electrospinning, which leads to a decrease in fiber diameter [34]. The porosity of the 3D printed-electrospun PCL/nHA/MWCNTs scaffolds was 75.78 ± 7.00%, and the porosity of PCL/nHA scaffolds and the pure PCL scaffolds was 73.44 ± 7.37% and 71.09 ± 8.97% (Table 2). Compared with PCL scaffolds, EDS analysis exhibited that simultaneous Ca and P peaks, and enhanced C signal, indicating the existence of Haps particles and the diffusion of MWCNTs in PCL/nHA/MWCNTs scaffolds (Fig. 3B). The absence of peaks at other locations proved that the solvent has been completely removed. The elemental distribution maps were shown in Fig. 3C, where purple, green, blue and yellow signals represented O, C, Ca and P, respectively. For pure PCL, no blue and yellow signals were presented, while blue and yellow signals were observed for PCL/nHA/MWCNTs composites, and the intensity of green signal was significantly enhanced, which was consistent with the above EDS spectrum analysis. In conclusion, we successfully prepared dual-scale 3D printed-electrospun PCL/nHA/MWCNTs scaffolds.

**Table 2.** The morphology and physical properties of the 3D printed-electrospun scaffolds

Sample	Electrospun	3D printing		Porosity (%)
	Fiber diameter (μm)	Filament width (μm)	Pore size (μm)	
PCL	1.47 ± 0.38	200.07 ± 19.89	504.54 ± 117.50	71.09 ± 8.97
PCL/nHA	1.74 ± 0.29	209.20 ± 15.81	494.89 ± 41.03	73.44 ± 7.37
PCL/nHA/MWCNTs	1.68 ± 0.62	239.45 ± 41.96	472.83 ± 59.79	75.78 ± 7.00

The mechanical properties of tissue-engineered scaffolds are significant characteristic parameters that determine their application [35, 36]. The mechanical properties of the scaffolds as bone substitutes are characterized by their compressive strength, which requires resistance to abrasion caused by movement or pressure from surrounding tissues [37]. The compression stress–strain curves were showed in Fig. 2B. The mechanical test results showed that the compression modulus of PCL/nHA/MWCNTs scaffold was 10.68 ± 0.24 MPa, which was higher than that of PCL/nHA scaffold (8.23 ± 0.10 MPa) and the pristine PCL scaffold (4.40 ± 0.09 MPa) (Fig. 2C). This may be due to the improvement in the compression modulus of composite caused by nHA and MWCNTs [24, 38]. The compression modulus of the scaffolds was found to increase with increase in nHA and MWCNTs content (see Supplementary Material, Fig. S1). The results showed that nHA and MWCNTs were uniformly dispersed in PCL matrix without agglomeration. MWCNTs appeared to establish strong links between nHA and PCL matrix. The compressive strength of the 3D printed-electrospun PCL/nHA/MWCNTs scaffold was close to that of cancellous bone (2–13 MPa) [39].

### The cytocompatibility of the biomimetic subchondral bone scaffold

**Figure 4.**
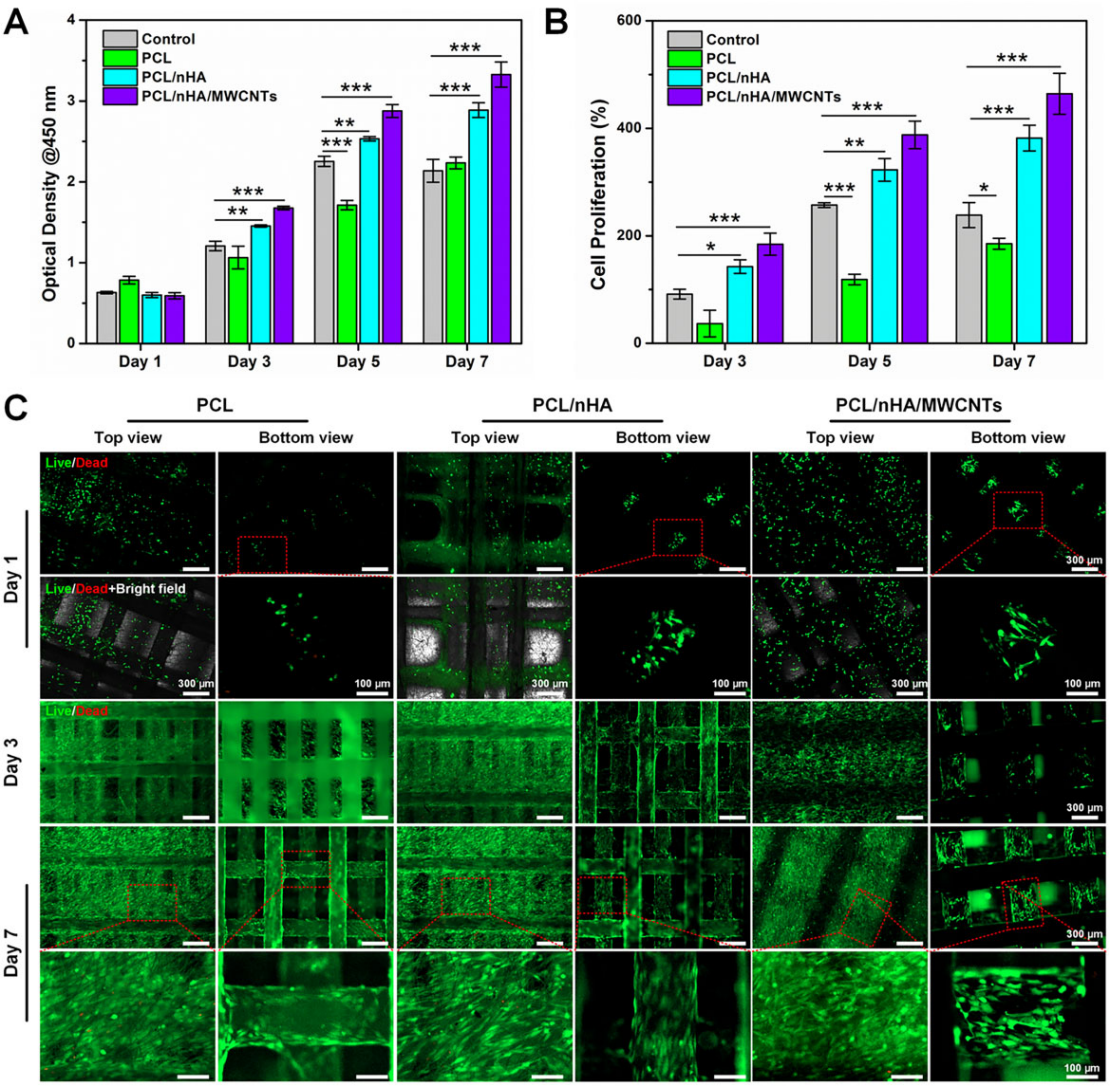
The cytocompatibility of the 3D printed-electrospun PCL, PCL/nHA and PCL/nHA/MWCNTs scaffolds. (**A**) The BMSCs viability using CCK-8 assay and (**B**) relative cell proliferation after 1, 3, 5 and 7 days of culture. (**C**) Live/dead staining of BMSCs cultured on the scaffolds at day 1, 3 and 7.

The internal structure of the scaffold is critical for cell migration and adhesion to the surface or interior of the scaffold. BMSCs were cultured on the 3D printed-electrospun PCL, PCL/nHA and PCL/nHA/MWCNTs scaffolds to assess cellular proliferation by CCK-8 assay at 1, 3, 5 and 7 days. BMSCs cultured in tissue culture plate were set as control group. As shown in Fig. 4A, there were no significant differences among the groups on day 1. With the increase of culture time, the optical density values increased, and PCL, PCL/nHA and PCL/nHA/MWCNTs showed an increasing trend of cell proliferation. As expected, BMSCs seeded on the PCL/nHA/MWCNTs scaffold exhibited the highest proliferation rate, reaching 464% at day 7 (Fig. 4B), and BMSCs proliferation in late stage was significantly faster than that in early stage.

Cell viability and density are much important for osteogenic differentiation. The Live/Dead staining assay also gave the same results as CCK-8. Fluorescence images exhibited that BMSCs survived and spread well on all scaffolds (Fig. 4C). However, some BMSCs shed through larger pores of the 3D scaffold during initial seeding, rather than sticking to the filaments. With the increase of culture time, due to the existence of electrospun fibers, the effective surface area for BMSCs to attach was increased and the BMSCs bridging between 3D printed filaments was promoted. Compared with PCL and PCL/nHA, there were fewer dead cells (red) and larger quantity of living cells (green) on PCL/nHA/MWCNTs membrane and 3D printed scaffold, indicating that BMSCs showed better activity on 3D printed-electrospun PCL/nHA/MWCNTs scaffolds.

**Figure 5.**
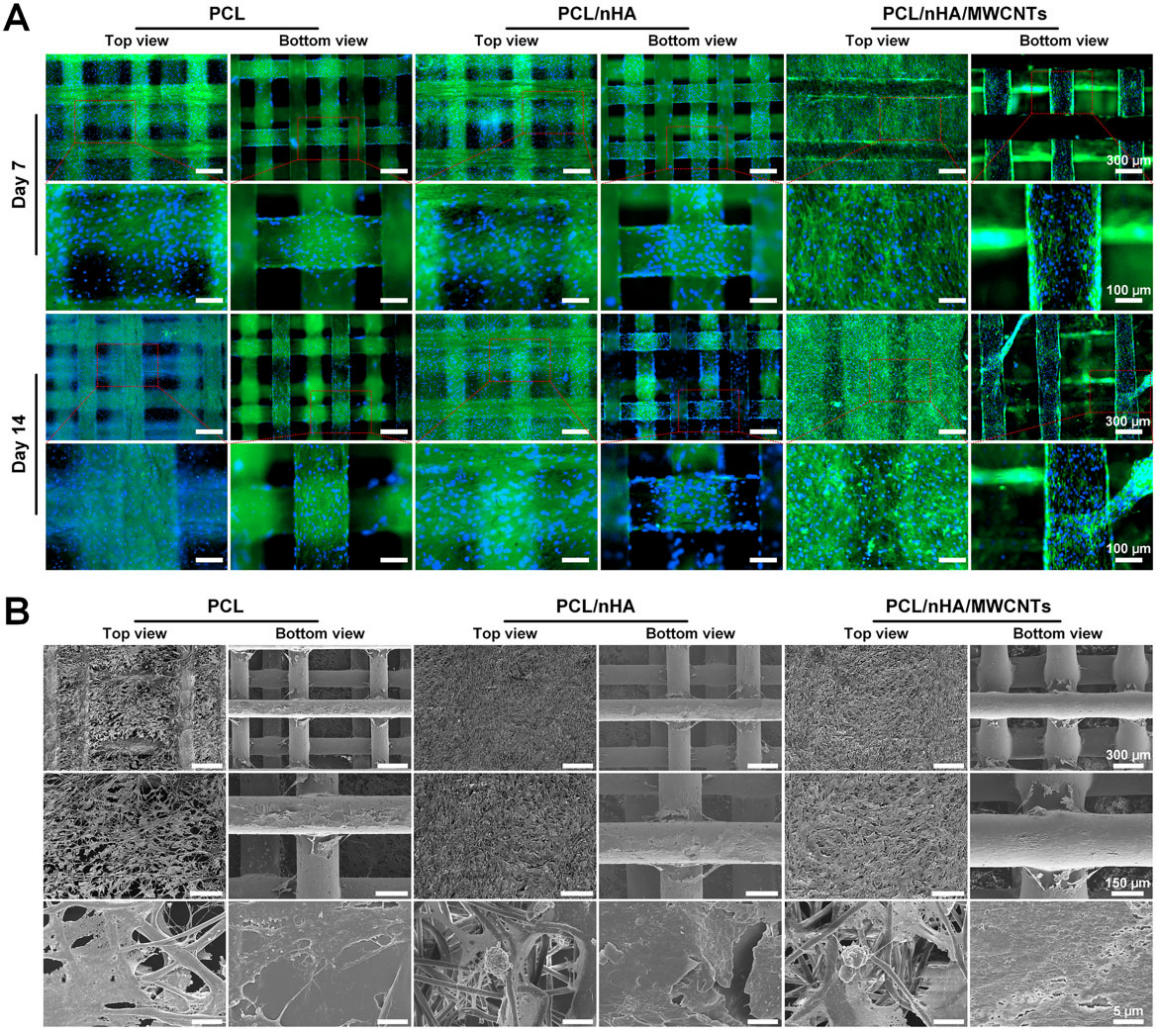
The morphology of BMSCS seeded on the 3D printed-electrospun PCL, PCL/nHA and PCL/nHA/MWCNTs scaffolds. (**A**) Cytoskeletal staining images of BMSCs seeded on scaffolds after 7 and 14 days of culture. (**B**) SEM images of BMSCs seeded on scaffolds after 14 days of culture.

Moreover, the top view of F-actin cytoskeleton staining revealed that BMSCs were anchored on the PCL/nHA/MWCNTs electrospun membrane and connected with each other, and the infiltration depth was significantly deeper than that of PCL and PCL/nHA membrane (Fig. 5A). Due to the presence of nanoscale electrospun fibers, the cytoskeletal structures of BMSCs were stretched and elongated, forming a spindle or polygon shape. The bottom view of cytoskeleton staining demonstrated that BMSCs were better distributed along the rough filaments of the 3D printed PCL/nHA/MWCNTs and PCL/nHA scaffolds than PCL (Fig. 5A). BMSCs attached to the surface of the 3D printed filaments grew along the printing direction on day 7 and bridged on day 14. The SEM images illustrated that more BMSCs closely attached and fully spread across the PCL/nHA/MWCNTs and PCL/nHA electrospun membrane than those seeded on the pristine PCL membrane after 14 days of culture (Fig. 5B). On the surface of 3D printed PCL/nHA/MWCNTs and PCL/nHA scaffold, BMSCs were fusiform with prominent filamentous pseudopods stretching into the surrounding pores. Consistent with cytoskeleton staining, the multi-scale topographical structure of the 3D printed-electrospun scaffolds served as physical cues affecting cell mechanotransduction, promoting BMSCs migration, growth and proliferation, and making BMSCs more easily adhere to the inside of the scaffolds. Cell adhesion is important for subsequent differentiation [40]. The incorporation of nHA and MWCNTs promoted the communication between MSCs and the interaction between MSCs and biomaterials [41]. Collectively, all of these results demonstrated that the 3D printed-electrospun PCL/nHA/MWCNTs scaffolds possessed excellent biocompatibility.

### The 3D printed-electrospun PCL/nHA/MWCNTs scaffolds accelerate osteogenic differentiation of BMSCs

Based on the aforementioned results of cytocompatibility, the 3D printed-electrospun PCL/nHA/MWCNTs scaffolds possessed the highest BMSCs proliferation rate and excellent activity. Subsequently, the osteogenic ability of PCL/nHA/MWCNTs scaffolds was evaluated with PCL as the control group.

**Figure 6.**
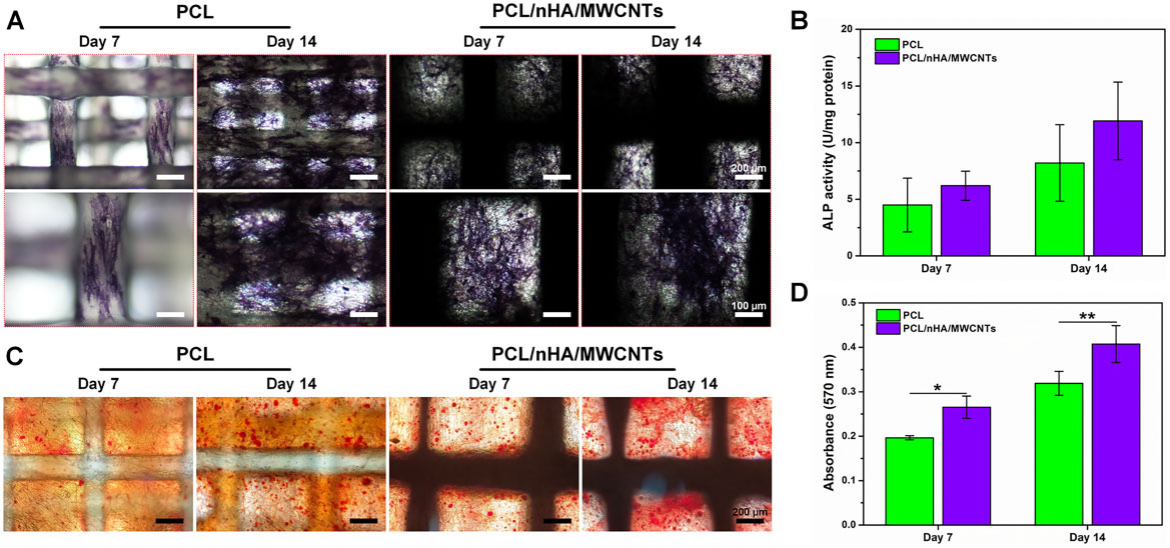
The osteogenic properties of BMSCs seeded on the 3D printed-electrospun PCL and PCL/nHA/MWCNTs scaffolds at days 7 and 14. (**A**) ALP staining images of BMSCs. (**B**) ALP activity of BMSCs normalized to total protein. (**C**) Alizarin Red staining images of BMSCs. (**D**) Quantitative analysis of Alizarin Red staining at OD 570 nm.

The osteogenic capacity of BMSCs was observed by ALP staining and ALP activity assay at days 7 and 14. ALP is an essential marker of early-stage osteogenic differentiation of MSCs mediated by biomaterials *in vitro* [42]. ALP staining (Fig. 6A) demonstrated that there were more ALP-positive cells in the nanoscale electrospun fibers and 3D printed filaments of PCL/nHA/MWCNTs group than in the PCL group, indicating that osteogenic differentiation was significantly enhanced. As displayed in Fig. 6B, the normalized ALP activity (6.20 ± 1.28 U/mg protein) of the 3D printed-electrospun PCL/nHA/MWCNTs group was higher than that of PCL group (4.49 ± 2.37 U/mg protein) on day 7, and was still obviously greater than that of PCL group on day 14, up to 11.92 ± 3.43 U/mg protein. This corresponds to a previous research that the introduction of MWCNTs played a leading role in the generation of ALP [41].

Additionally, ARS Staining was performed to detect ECM mineralization after 7 and 14 days of culture. As detected, both groups exhibited very weak ARS staining on day 7 (Fig. 6C). Compared with the PCL group, the de-staining level of ARS in the PCL/nHA/MWCNTs scaffold was the highest on day 14, confirming that the existence of Haps particles within the 3D printed-electrospun PCL/nHA/MWCNTs scaffold. The calcium-rich Haps particles were dyed dark red and evenly integrated throughout the PCL/nHA/MWCNTs construct, further facilitating calcium deposition. As reported in a study, the anisotropic characteristic of the dual-scale scaffolds also seem to facilitate the stimulation of osteogenic differentiation [43]. Quantitative analysis of calcium deposition was shown in Fig. 6D. The mineralization capacity of PCL/nHA/MWCNTs group was statistically higher than that of PCL group. The osteoblastic differentiation of BMSCs produced calcium, which then bound to proteins in the ECM to mineralize it. Thus, the 3D printed-electrospun PCL/nHA/MWCNTs scaffolds expedited and promoted the mineralization process.

**Figure 7.**
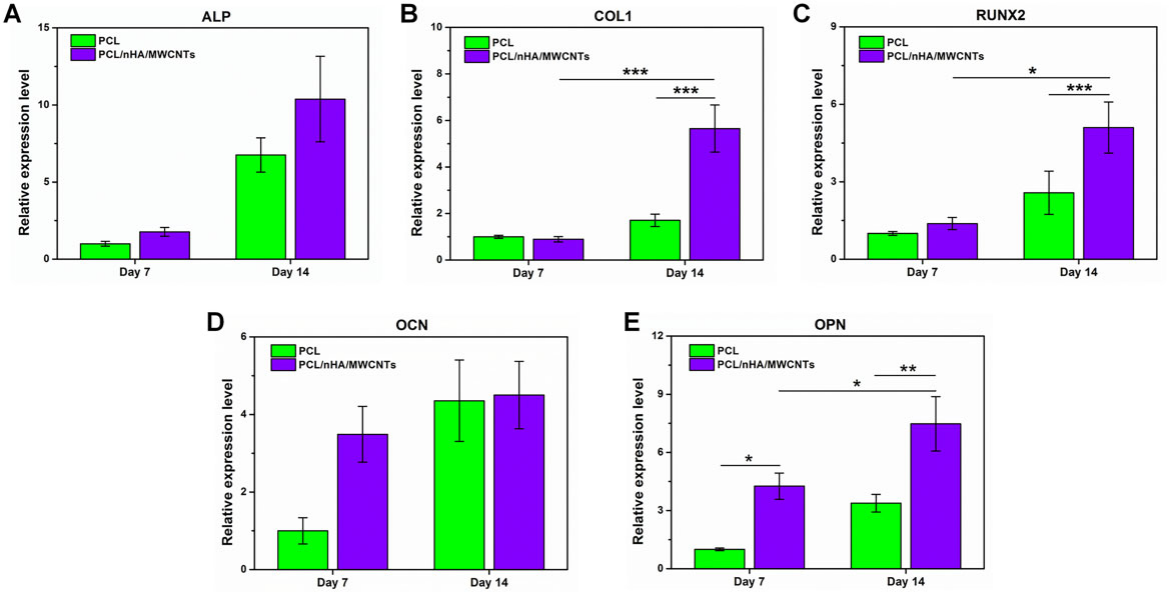
The expression of osteogenic marker genes in BMSCs seeded on the 3D printed-electrospun PCL and PCL/nHA/MWCNTs scaffolds determined by RT-PCR tests at day 7 and 14. (**A**) ALP, (**B**) COLI, (**C**) RUNX2, (**D**) OCN and (**E**) OPN.

**Figure 8.**
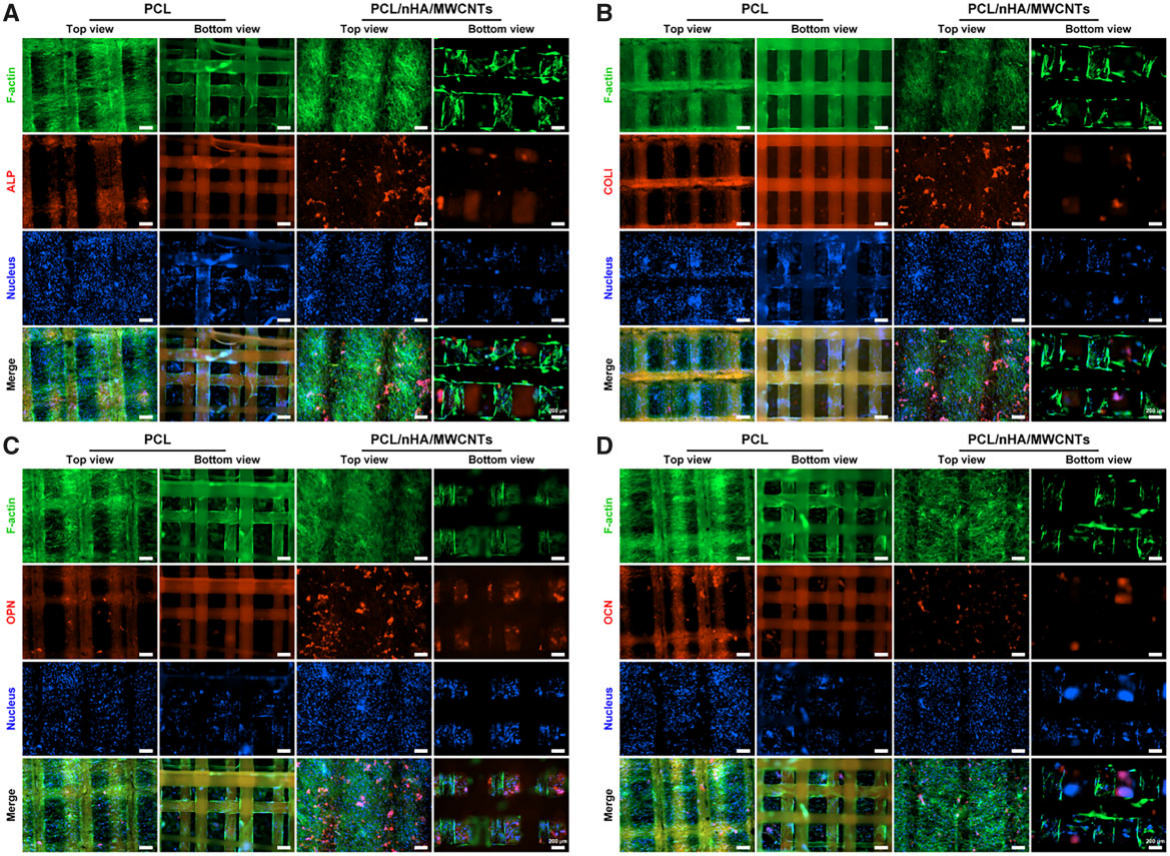
Immunofluorescence staining images of the osteogenic marker proteins secreted by BMSCs on the 3D printed-electrospun PCL and PCL/nHA/MWCNTs scaffolds after 14 days of culture. (**A**) ALP, (**B**) COL1, (**C**) OPN and (**D**) OCN. The scale bar is 200 μm.

Subsequently, qRT-PCR analysis was performed on days 7 and 14 to detect the regulation of gene expression and verify osteogenesis at the gene expression level (Fig. 7). All osteogenic related genes presented an apparent upregulation with the increase of culture time in both groups. On day 7, the PCL/nHA/MWCNTs group showed greater mRNA expression of ALP, RUNX2, OCN and OPN than PCL group. ALP, RUNX2 and COL1 are early-stage osteogenic expression genes, where ALP represents proliferation, and RUNX2 expression stimulates the activity of COL1 promoter fragment and increases matrix deposition [44–46]. On day 14, the expression levels of ALP, COL1 and RUNX2 were 1.54, 3.31 and 1.98 times higher in the PCL/nHA/MWCNTs group than in the PCL group, respectively. OCN and OPN are genes expressed in the late stage of osteogenic differentiation, which are crucial to the formation, metabolism and mineralization of bone matrix [47, 48]. On day 14, the expression levels of OCN and OPN were 1.03 and 2.21 times higher in the PCL/nHA/MWCNTs group than in the PCL group, respectively. Correspondingly, immunofluorescence images on the 14th day further confirmed that BMSCs on PCL/nHA/MWCNTs scaffolds secreted much more of osteogenic marker proteins, i.e. ALP, COL1, OPN and OCN, than those on PCL scaffolds (Fig. 8). Collectively, these results indicated that PCL/nHA/MWCNTs scaffold presented the most positive impact on the osteogenic differentiation of BMSCs *in vitro*.

## Conclusion

In this study, the 3D printed-electrospun PCL/nHA/MWCNTs scaffolds were successfully constructed by electrospinning combined with layer-by-layer 3D printing. The upper nanoscale electrospun membrane was tightly coupled with the lower 3D-printed scaffold to prevent stratification. The resulting dual-scale PCL/nHA/MWCNTs scaffold was compounded from a dense layer of disordered nanospun fibers and a porous 3D-printed scaffold layer for enabling and promoting the ingrowth of subchondral bone. Herein, the biomimetic PCL/nHA/MWCNTs scaffolds enhanced cell seeding efficiency and allowed for higher cell–cell interactions that supported BMSCs adhesion and proliferation, subsequently promoted osteogenic differentiation *in vitro*. Together, this study provides a functional biomimetic subchondral bone scaffold, which lays a foundation for subsequent studies, such as immobilizing different bioactive factors on nanoscale electrospun fibers and 3D-printed filaments, and combining with hydrogel to promote osteochondral tissue regeneration *in vivo*, particularly subchondral bone repair.

## Supplementary Material

rbac104_Supplementary_Data
