# Supplementary material for: 3D printed-electrospun PCL/hydroxyapatite/MWCNTs scaffolds for the repair of subchondral bone
Source: Regen Biomater. 2022 Dec 14;10:rbac104. doi: 10.1093/rb/rbac104 (PMC9847519; doi:10.1093/rb/rbac104)
Supplement: rbac104_Supplementary_Data [file rbac104_supplementary_data.docx]

**Supplementary Material**

**3D printed-electrospun PCL/Hydroxyapatite/MWCNTs scaffolds for the repair of subchondral bone**

Yanyan Cao^1,2^, Lei Sun^2,3^, Zixian Liu^2,3^, Zhizhong Shen^2,4^, Wendan Jia^2,4^, Peiyi Hou^2,4^, Shengbo Sang^2,3,^*

^1^College of Information Science and Engineering, Hebei North University, Zhangjiakou, 075000, China

^2^Shanxi Key Laboratory of Micro Nano Sensors & Artificial Intelligence Perception, College of Information and Computer, Taiyuan University of Technology, Taiyuan, 030024, China

^3^Key Lab of Advanced Transducers and Intelligent Control System of the Ministry of Education, Taiyuan University of Technology, Taiyuan, 030024, China

^4^Shanxi Research Institute of 6D Artificial Intelligence Biomedical Science, Taiyuan, 030031, China

* Corresponding authors.

Shengbo Sang, Ph.D., E-mail: sunboa-sang@tyut.edu.cn


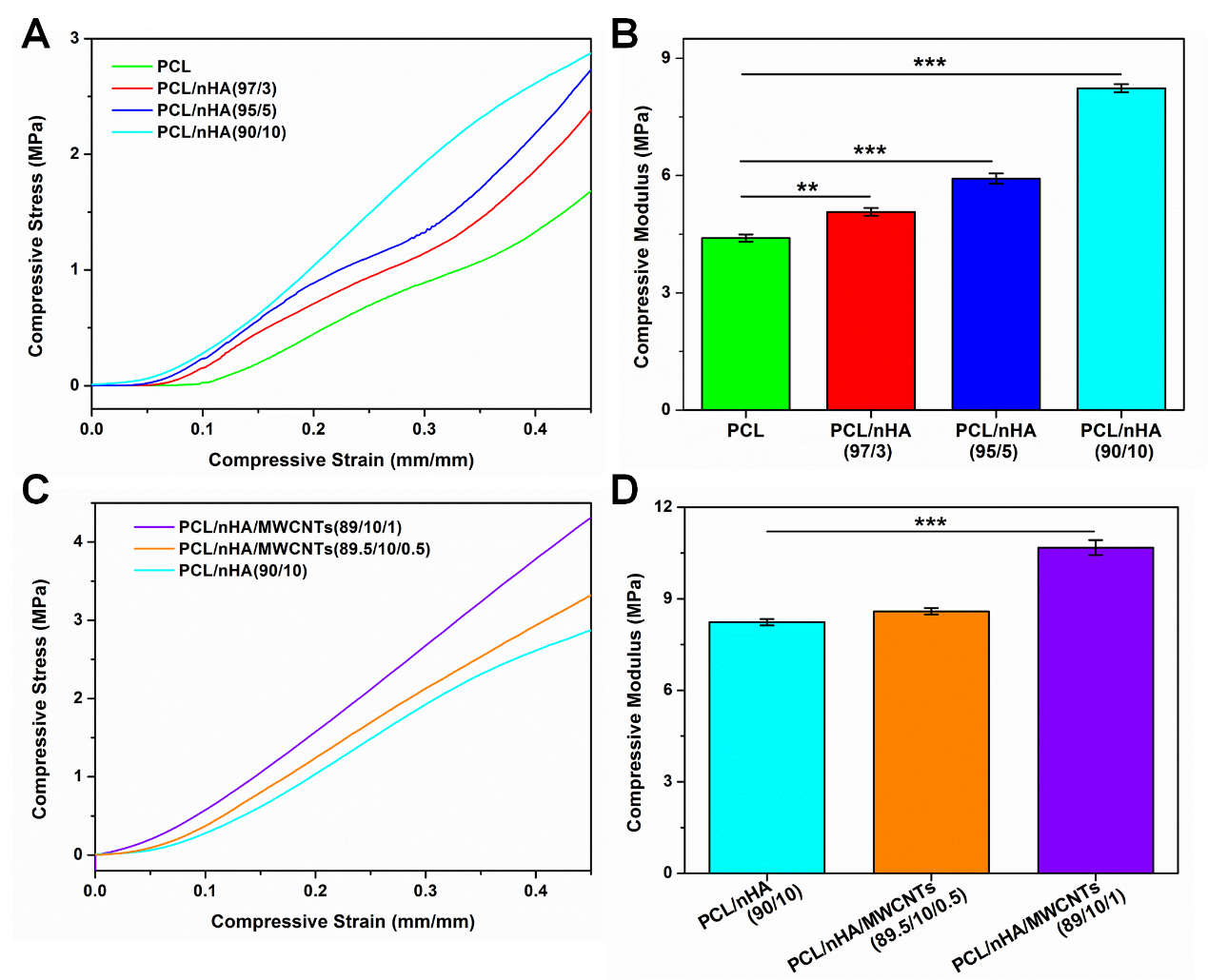


**Fig.S1** A) Compressive stress-strain curves and B) compressive modulus of the 3D printed-electrospun PCL/nHA scaffolds (nHA content = 0, 3, 5 and 10 wt%). C) Compressive stress-strain curves and D) compressive modulus of the 3D printed-electrospun PCL/nHA/MWCNTs scaffolds (MWCNTs content = 0, 0.5 and 1 wt%).
